# Philosophical analysis of the Recovery College learning model: characterization and connections to learning theories

**DOI:** 10.3389/fpsyt.2025.1613074

**Published:** 2025-07-28

**Authors:** Galaad Lefay, Catherine Briand, Anick Sauvageau, Marie-Josée Drolet, Brigitte Vachon, Francesca Luconi, Aliki Thomas, Juliette Nadeau-Tremblay

**Affiliations:** ^1^ Department of occupational therapy, University of Quebec at Trois-Rivières, Trois-Rivières, QC, Canada; ^2^ Research Center of Institut universitaire en santé mentale de Montréal, Montréal, QC, Canada; ^3^ Centre de recherche en éthique (CRÉ), Université de Montréal, Montréal, QC, Canada; ^4^ Institut d’éthique appliquée (IDÉA), Université Laval, Québec city, QC, Canada; ^5^ School of Readaptation, University of Montreal, Montréal, QC, Canada; ^6^ Continuing Professional Development, McGill School of Medicine, McGill University, Montréal, QC, Canada; ^7^ Center for Interdisciplinary Research in Rehabilitation of Metropolitan Montreal, Montréal, QC, Canada

**Keywords:** recovery college, learning theories, philosophical analysis, epistemic justice, mental health

## Abstract

**Introduction:**

The Recovery College (RC) model of learning is an innovative approach that originated in the UK in 2009 and has rapidly expanded, boasting over 130 locations in 22 countries by 2021. Grounded in the coproduction and recognition of various types of knowledge (clinical, experiential, theoretical), it fosters mental health, well-being, and social inclusion by bringing together diverse participants to learn collaboratively. However, despite its originality, few in-depth studies have examined its theoretical foundations, particularly its connection to social constructivism, which emphasizes collaborative learning and social interaction. A theoretical and philosophical analysis of this learning model would enhance our understanding of its mechanisms of action and enrich the pedagogical practices of RCs while considering adaptations for other contexts.

**Objectives:**

This study aims to define and characterize the Recovery College learning model and identify its connections with the key learning theories through a theoretical and philosophical analysis.

**Methodology:**

The study employs a hermeneutic philosophical approach consisting of six steps: 1. define and characterize the RC learning model, 2. identify, define, and describe the key learning theories, 3. select the perspectives and questions for philosophical analysis, 4. analyze the RC learning model through the chosen philosophical perspectives and questions, 5. identify the philosophical connections with the key learning theories, and 6. validate the analysis process.

**Results:**

The analysis identified five mechanisms of action, nine key principles of RC and four operations. RC integrates important concepts from social constructivism, cognitive constructivism, andragogy, and transformative learning, emphasizing collaborative, experiential, autonomous, and context-driven knowledge development. Philosophical analyses from epistemological, ethical, and political perspectives highlight RC’s role in addressing epistemic justice, power relations, and inclusive learning spaces.

**Discussion:**

The Recovery College proposes an innovative approach that values the plurality of knowledge (clinical, experiential, theoretical) to redress epistemic injustices and rebalance relationships among different types of knowledge. Creating safe and egalitarian epistemic spaces supports inclusive learning aligned with principles of equity, diversity, and inclusion. Its ethico-political stance addresses systems of oppression (ableism, ageism, sanism) by bringing together diverse individuals in equality, thereby deconstructing stigma and prejudice. This approach, rooted in collaborative learning theories, transforms individuals and systems while enriching educational practices.

## 1 Introduction

Since its emergence, the Recovery College (RC) has established itself as a unique educational and mental health initiative, attracting increasing interest worldwide (1, 2). Rooted in the recovery-oriented transformation of mental health services, the RC is recognized as one of the key practices supporting personal recovery (3). The Implementing Recovery through Organisational Change (ImROC) guide explicitly identifies the establishment of RCs as one of the ten key challenges for services committed to recovery-oriented change (1). Designed to promote well-being, mental health, and social inclusion, the RC is based on an innovative learning model that encompasses both theoretical and practical aspects. Launched in the United Kingdom in 2009, the RC has experienced remarkable expansion. By 2021, over 130 RCs had been identified in 22 countries (2). Some Recovery Colleges are integrated into formal healthcare systems, for instance, many in the UK operate within the National Health Service (NHS) and are typically co-developed and co-delivered by people with lived experience of mental health challenges and professionals. This learning model unites various participants (referred to as learners): individuals living with mental illness, health professionals, managers, and other citizens who learn in an environment that encourages exchange and mutual understanding (4). The essence of the RC lies in the diversity of knowledge, clinical, experiential, or theoretical, and in their equal recognition. According to Toney et al. (5), this process fosters a shared understanding and strengthens the sense of inclusion. The coproduction of knowledge, the diversity of learners, and the acknowledgment of lived experiences are fundamental principles of this model and legitimate sources of knowledge (1). Evidence suggests that learners often report increased well-being, improved self-confidence, greater social inclusion, and, in some cases, reduced use of clinical services (5, 6).

Despite the richness and variety of existing contributions to RC, these primarily focus on its development, implementation, and effects on learners (7). The theoretical foundations of this learning model remain insufficiently explored, creating a significant gap in our understanding of its functioning. An in-depth analysis of the conceptual foundations would deepen our comprehension of the learning dynamics specific to RC, illuminating the mechanisms that underlie knowledge coproduction and interactions among learners from diverse backgrounds. Works such as Mcgregor et al. (8), which draws on social constructivism, initiated this approach by highlighting the importance of coproduced training and mutual learning in shaping educational experiences within RCs. However, the literature does not fully articulate the connections between the fundamental principles of the RC learning model and the key learning theories that could elucidate how it functions.

In this context, a theoretical and philosophical analysis contributes to a deeper understanding of the RC learning model (9, 10). Engaging this learning model in dialogue with key learning theories makes it possible to explore how core principles, such as coproduction, collaborative learning and the valuing of experiential knowledge, fit into a broader educational paradigm. Such a reflection can also shed light on how the RC learning model adapts and transposes these theories to non-traditional contexts while contributing to developing innovative pedagogical frameworks tailored to learners’ needs. Analyzing the theoretical and philosophical foundations of RC could therefore enrich our understanding of this model.

This article aims to characterize the Recovery College learning model and identify its connections with key learning theories through a theoretical and philosophical analysis. To this end, three specific objectives are defined:

1. Define and characterize the RC learning model.
2. Identify the key learning theories related to the RC learning model.
3. Analyze the connection between the RC learning model and these learning theories.

## 2 Method

To achieve the study’s objectives, a theoretical and philosophical analysis of a hermeneutical nature based on the works of Gadamer (9) and Paillé and Mucchielli (10) was conducted. According to Gadamer (9), hermeneutics is a philosophical approach to interpretation that highlights the dynamic and contextual understanding of texts. This approach allows for a logical and semantic analysis of content while taking into account the context in which texts develop (10). To do this study, a six-step method is proposed (see **Table 1**). The six-step procedure (**Table 1**) was synthesized by the authors from hermeneutic principles articulated by Gadamer (9) and the iterative coding strategy described by Paillé and Mucchielli (10).

**Table 1:** Six-step method for theoretical and philosophical analysis.

Step	Description
1	Define and characterize the Recovery College learning model
2	Identify, define and characterize the key learning theories
3	Choosing perspectives and questions for philosophical analysis
4	Analyze the Recovery College learning model through philosophical perspectives and questions
5	Identify connections with the selected learning theories
6	Validate the analysis process

### 2.1 Step 1: Define and characterize the RC learning model

#### 2.1.1 Identifying texts for analysis

The first corpus is based on a literature review following the Systematized review method conducted by Briand et al. (7). The bibliographic search was conducted on the MEDLINE and Scopus databases and included peer-reviewed studies published between 2012 and 2023. The keywords “Recovery College*” and “Recovery education* center” were used. A total of 80 articles were retained after eliminating duplicates and irrelevant articles. Finally, the first briefing paper on RC by Perkins et al. (1) was added for its relevance, bringing the total number of texts processed to 81.

#### 2.1.2 Sorting of texts according to inclusion and exclusion criteria

**Figure 1:**
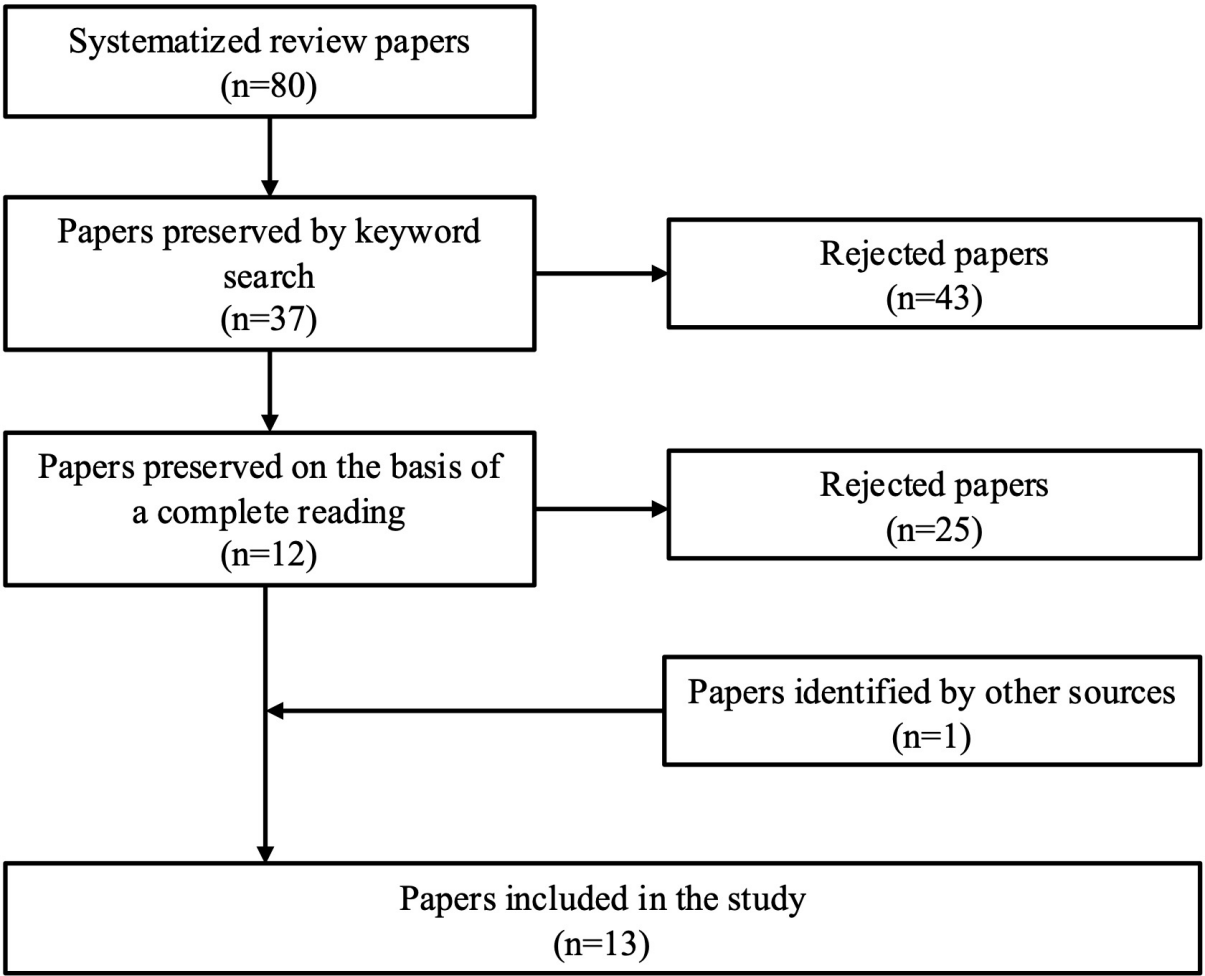
PRISMA.

The 81 texts previously identified were considered and entered into the EndNote bibliographic reference management software. An initial selection was made based on a keyword search. Texts containing the following keywords were retained: “mechanisms of action”, “mechanism of change”, “transformative power”, “key principle,” and “critical dimensions”. A pilot scan of RC literature showed that authors employ heterogeneous terminology when describing how RC works. The five terms chosen capture this lexical diversity and ensured a comprehensive retrieval of passages dealing with the Recovery College learning model. After this text selection (n=38), two authors (GL and CB) conducted a blind selection in parallel according to three specific criteria: 1) the study aims to explore the mechanisms of action of Recovery College (RC), 2) the results provide new insights into the model’s mechanism of action, and 3) these insights are discussed in depth. A total of 13 texts were retained for analysis. **Figure 1** illustrates the selection process according to PRISMA guidelines, while **Table 2** displays the selected texts.

**Table 2:** RC learning model: corpus of texts in chronological order.

Perkins R, Repper J, Rinaldi M, Brown H. Recovery colleges. London: ImROC briefing paper 1 (2012). Mcgregor J, Repper J, Brown H. The college is so different from anything I have done”. A study of the characteristics of Nottingham Recovery College. *J Ment Health Training Educ Pract*. (2014) 9:3–15. doi: 10.1108/JMHTEP-04-2013-0017 Perkins R, Repper J. When is a “recovery college” not a “recovery college”? *Ment Health Soc Inclusion*. (2017) 21:65–72. doi: 10.1108/MHSI-02-2017-0005 Shepherd G, Mcgregor J, Meddings S, Roeg W. Recovery colleges and coproduction. In: Slade M, Oades L, Jarden A, editors. Wellbeing, recovery and mental health. Cambridge: Cambridge University Press (2017). p. 181-93. Sommer J, Gill K, Stein-Parbury J. Walking side-by-side: Recovery Colleges revolutionising mental health care. *Ment Health Soc Inclusion*. (2018) 22:18–26. Toney R, Elton D, Munday E, Hamill K, Crowther A, Meddings S, et al. Mechanisms of action and outcomes for students in recovery colleges. *Psychiatr Serv*. (2018) 69:1222–9. doi: 10.1176/appi.ps.201800283 Crowther A, Taylor A, Toney R, Meddings S, Whale T, Jennings H, et al. The impact of Recovery Colleges on mental health staff, services and society. *Epidemiol Psychiatr Sci*. (2019) 28:481–8. doi: 10.1017/S204579601800063X Muir-Cochrane E, Lawn S, Coveney J, Zabeen S, Kortman B, Oster C. Recovery college as a transition space in the journey towards recovery: An Australian qualitative study. *Nurs Health Sci*. (2019) 21:523–30. doi: 10.1111/nhs.12637 Toney R, Knight J, Hamill K, Taylor A, Henderson C, Crowther A, et al. Development and evaluation of a recovery college fidelity measure. *Can J Psychiatry*. (2019) 64:405–14. doi: 10.1177/0706743718815893 Reid N, Khan B, Soklaridis S, Kozloff N, Brown R, Stergiopoulos V. Mechanisms of change and participant outcomes in a Recovery Education Centre for individuals transitioning from homelessness: a qualitative evaluation. *BMC Public Health*. (2020) 20:497. doi: 10.1186/s12889-020-08614-8 Bester KL, Mcglade A, Darragh E. Is co-production working well in recovery colleges? Emergent themes from a systematic narrative review. *J Ment Health Training Educ Pract*. (2021) 17:48–60. doi: 10.1108/JMHTEP-05-2021-0046 Thompson H, Simonds L, Barr S, Meddings S. Recovery colleges: long-term impact and mechanisms of change. *Ment Health Soc Inclusion*. (2021) 25:232–42. doi: 10.1108/MHSI-01-2021-0002 Doroud N, King A, Zirnsak TM, Brasier C, Hall T, Jordan H, et al. Creating “an oasis of hope, inclusion and connection”: students and stakeholders’ experiences of a pilot Recovery College. *J Ment Health*. (2023) 33:92–100. doi: 10.1080/09638237.2023.2245881

In this study, the learning model is examined through the lens of its mechanisms of action, which allows for comprehension of how the principles and objectives of the model will translate into effects. Mechanisms of action are defined as dynamic processes central to the RC learning model that catalyze transformations observed in learners (11, 12). These mechanisms of action influence and reshape learners’ reasoning and behaviours in response to the resources and activities proposed in the intervention (13). Often called the “black box” components, mechanisms of action elucidate how and why an intervention functions in various contexts and with diverse individuals. They establish the connection between the intervention and its outcomes (13). While not necessarily directly observable, they can be deduced from the effects they produce (14).

#### 2.1.3 Analysis of the first corpus

For each article selected, the conceptual framework, results and discussion sections were analyzed to define and characterize the RC learning model. For this study, a multi-level reading, inspired by the work of Atsbury and Leeuw (13), was carried out:

1. The first level, situated at the macroscopic scale, encompasses overarching principles. As general concepts, these principles influence how training courses within RCs are conceived and developed.
2. The second level, situated at the mesoscopic scale, comprises the operations. Presented in the form of infinitive action verbs, operations facilitate the concrete adoption of the principles.
3. The third level, situated at the microscopic scale, pertains to the pedagogical tools and strategies employed during training courses. As the most applied dimension of training, it directly precedes the ‘outcomes’—that is, the effects of RC training courses on learners. This level was not analyzed in the present study due to insufficient documentation in the source texts.

The mechanisms of action represent the transversal process between these three levels, expressing the dynamic transformation of a principle into a concrete pedagogical strategy.

To characterize the mechanisms of action according to this reading grid, a double-blind analysis process was conducted with the first five texts (38.5% of the total). This process allowed the two authors (GL and AS) to independently thematize the data using NVivo 14 software, based on the two reading levels (principles and operations). Regular meetings were held to resolve disagreements and harmonize the codebook, ensuring a consistent approach to analyzing the subsequent texts. From the sixth text onward, the first author (GL) continued the analysis independently, which was reviewed and validated several times by two other authors specializing in the RC learning model (AS and CB). A research journal was maintained throughout the process to record ideas, potential connections, and relevant observations. Lastly, similar ideas were merged to finalize the codebook. The codebook served to organize and define the codes and categories resulting from the analysis, thus ensuring the rigour and transparency of the process (10). Definitions were refined based on various text extracts, and style was harmonized according to the main categories: mechanisms, principles, and operations.

### 2.2 Step 2: Identify, define and characterize the key learning theories

#### 2.2.1 Identifying learning theories and texts for analysis

First, it identified the learning theories that reflect the mechanisms of action, principles, and operations noted in the analysis of the first corpus. To this end, three authors (BV, FL, AT), who are experts in educational theories, explored the definition and characterization of the RC learning model. They identified five main theories: 1. social constructivism, 2. cognitive constructivism, 3. transformative learning, 4. Andragogy, and 5. situated learning. Experiential learning was considered, but its core propositions (learning through experience, reflection, and abstraction) are already encompassed within social and cognitive constructivism and therefore would have introduced redundancy. They then highlighted several foundational texts of these theories, which were analyzed by the two authors (GL, AS) to reveal their key principles. A total of 15 texts were selected and are presented in **Table 3**.

**Table 3:** Learning theories: corpus of texts in chronological order.

Piaget J. *The origins of intelligence in children*. Cook M, translator. New York: International Universities Press (1952). Festinger L. *A theory of cognitive dissonance*. Stanford, CA: Stanford University Press (1957). Berger PL, Luckmann T. *The social construction of reality: A treatise in the sociology of knowledge*. Garden City (NY): Anchor Books (1966). Aronson E. Theory of cognitive dissonance: A current perspective. In: Berkowitz L, editor. Advances in experimental social psychology. New York: Academic Press (1969). Vygotsky LS. *Mind in society: the development of higher psychological processes*. Cambridge, MA: Harvard University Press (1978). Knowles MS. The modern practice of adult education: from pedagogy to andragogy (2nd ed). Englewood Cliffs, NJ: Cambridge Adult Education (1980). Brown JS, Collins A, Duguid P. Situated cognition and the culture of learning. *Educ Researcher*. (1989) 18:32–42. doi: 10.3102/0013189X018001032 Mezirow J. *Transformative dimensions of adult learning*. San Francisco, CA: Jossey-Bass (1991). Steffe LP, Gale JE (Eds.). *Constructivism in education*. Hillsdale, NJ: Lawrence Erlbaum Associates (1995). Von Glasersfeld E. A constructivist approach to teaching. In: Steffe LP & Gale SJE, editors. Constructivism in education. Hillsdale, NJ: Lawrence Erlbaum Associates (1995), p. 3-15. Greeno JG. On claims that answer the wrong questions. *Educ Researcher*. (1997) 26:5–17. doi: 10.3102/0013189X026001005 Lave J, Wenger E. *Communities of practice: Learning, meaning, and identity*. Cambridge (UK): Cambridge University Press (1998). Mezirow J. *Learning as transformation: critical perspectives on a theory in progress*. San Francisco, CA: Jossey-Bass (2000). Goodrich RA. Vygotsky in perspective. *Philos Psychol*. (2013) 27:926-30. doi: 10.1080/09515089.2013.775634 Merriam SB, Bierema LL. *Adult learning: linking theory and practice*. San Francisco, CA: Jossey-Bass (2014). Hoggan CD. Transformative learning as a metatheory: Definition, criteria, and typology. *Adult Educ Q*. (2016) 66:57–75. doi: 10.1177/0741713615611216

#### 2.2.2 Analysis of the second corpus

The analysis of the foundational texts of learning theories was conducted in four stages. A preliminary reading of the corpus helped identify the relevant chapters. The chapters were subsequently reviewed to identify and define the key concepts of the theories discussed. To enrich and refine these definitions, additional works that comment on, discuss, or critically analyze the original texts were consulted as needed and incorporated when deemed relevant (n=2). To do that, a supplementary search in databases was done. Lastly, a codebook was created to synthesize the definitions of the theories and their key principles. The four stages were executed in parallel by two authors (GL, AS), who held regular meetings to address discrepancies and harmonize the codebook. A research journal was maintained throughout the analysis to document significant ideas, observations, and connections.

### 2.3 Step 3: Choosing perspectives and questions for philosophical analysis

Three perspectives for philosophical analysis were selected: epistemological, ethical, and political. This selection emerged from discussions among four of the authors aimed at ensuring a rich analysis while avoiding redundancy (GL, CB, MJD, BV).

#### 2.3.1 Epistemological perspective

According to Seymour (15) and Drolet (16), epistemology is a critical branch of philosophy concerned with the study of knowledge. It questions the nature, sources and criteria of knowledge, how we can learn and the limits of what we can know. As part of this study, several questions related to epistemology guided the analysis of the texts: What is considered knowledge? How is new knowledge developed and transmitted? What are the knowledge development objectives in the RC learning model?

#### 2.3.2 Ethical perspective

Ethics, as defined by Seymour (15), is a philosophical discipline that examines moral principles and values. It aims to study what guides actions and decisions through reflection on moral behaviour and ethical judgments. Ethics involves rational and critical reflection on how principles and values should guide our lives together (16). Within the context of this study, an ethical perspective raises several questions: What values underlie the RC learning model? How are these values embodied and transmitted? How do they influence learning and the learning space?

#### 2.3.3 Political perspective

Politics examines concepts and theories related to governance, social justice, rights, freedom, and authority (15). It aims to understand the relationships between individuals and organizations and how social actions affect and reshape society. Politics is closely tied to the concept of power and its exercise and contestation within society to reflect and implement ethical values and perspectives (15, 17). In the context of this study, a political perspective raises the following questions: How is power distributed among people in learning spaces? What strategies are employed within the RC learning model to promote equality among individuals, including learners?

### 2.4 Step 4: Analyze the RC learning model through philosophical perspectives and questions

At this stage, the author (GL) addressed each philosophical question based on the definitions of the RC learning model’s mechanisms of action, principles, and operations. These responses provide a clear philosophical positioning of the learning model and serve as a preliminary step toward connecting it with the key learning theories.

### 2.5 Step 5: Identifying connections with the key learning theories

It is important to distinguish between learning theories and learning models, while recognizing their complementary nature. A learning theory offers a general explanatory framework for how individuals process knowledge, often grounded in philosophical principles. It provides a broad understanding of the mechanisms of action underlying learning

A learning model is the practical extension of this theory. It applies and structures theoretical principles, adapting them to specific pedagogical contexts. As Willett (18) points out, the model does not seek to compete with the theory but to concretize its concepts to meet specific needs while remaining flexible and evolving.

This study explores the RC learning model to highlight connections with the learning theories selected.

### 2.6 Step 6: Validate the analysis process

To ensure scientific rigor, regular meetings were held to compare the analyses and enhance inter-rater reliability. The analyses were presented to all authors at each stage for validation, redirection, and refinement.

## 3 Results

The results are organised into three sections. The first section describes the first corpus of texts analysed and outlines the RC learning model. The second section discusses the subsequent corpus of texts and outlines the learning theories considered in this study. Finally, the third section provides a theoretical and philosophical analysis of the RC learning model and identifies its connections to learning theories.

### 3.1 Definition and characterization of the RC learning model

The first corpus consists of 13 studies published between 2012 and 2023, conducted primarily in the United Kingdom but also in Australia (19, 20) and Canada (21). The types of learners vary across studies, with RCs being targeted towards both the general population (5, 22) and more specific groups, such as health professionals (23) and individuals experiencing homelessness (21). This diversity enables the examination of different forms and contexts of implementation, highlighting the flexibility of the RC learning model and the range of issues encountered based on learner profiles.

Early work (1, 8) primarily described the foundations of the RC learning model and highlighted the impact as perceived by learners. By 2017, several studies (23, 24) began to focus more specifically on the components likely to foster a lasting impact on learners. From 2018 to 2019, the focus expanded to understanding the mechanisms of action through finer-grained qualitative analysis (20, 22, 25), particularly emphasizing the role of coproduction and collaboration between learners and other interested parties. More recent publications (21, 26–28) concentrate more on measuring the sustainability of these effects and examining their implications for healthcare systems, while also consolidating knowledge from earlier phases.

Regarding methodology, the initial studies (1, 8) are primarily descriptive, often receiving moderate quality scores (75% and below). These scores were assessed according to the methodology outlined by Kmet et al. (29) in the systematic review by Briand et al. (7). Research published from 2017 onwards (e.g., 5, 19, 25) adopts more structured qualitative approaches, with some conducting systematic reviews to compare their findings with the existing literature (26). Lastly, the most recent studies (22, 28) are achieving quality assessments classified as “very good” or even “excellent,” incorporating measures of fidelity (5) and placing particular emphasis on data triangulation. This improvement in quality reflects a commitment to creating a more robust and relevant body of knowledge to inform practice and policy in this area.

**Table 4** offers a synthesized and interpretive summary of the Recovery College (RC) learning model, capturing its structural and conceptual foundations. Drawing from the analysis of a carefully selected corpus of 13 studies, the model is articulated through five mechanisms of action, nine guiding principles, and four core operations. Together, these elements illustrate how the RC approach fosters transformative learning, grounded in collaboration, inclusion, and epistemic justice. This table serves not only as a descriptive framework but also as an interpretive lens, offering insight into how the RC model translates philosophical ideals into concrete educational practices.

**Table 4:** Characterization of the mechanisms of action, principles and operations of the RC learning model.

Category	Key elements	Authors	Definition
Mechanisms of Action	Transformative^1^ process of principles into outcomes, which can take different paths depending on the context.		
	Process of personal development and transformation	Perkins et al. (1) McGregor, Repper & Brown (8) Perkins, Repper (23) Toney et al. (25) Muir et al. (20) Toney et al. (5) Reid et al. (21) Thompson et al. (27) Doroud et al. (28)	Fosters personal and/or professional growth. It offers a space for transformation (competency development) and identity transition (normalized and positive) for learners.
	Process of emergence of different relationships	Perkins et al. (1) McGregor, Repper & Brown (8) Sommer, Gill & Stein-Par bury (19) Toney et al. (25) Crowther et al. (22) Reid et al. (21) Thompson et al. (27)	Fosters new forms of relationships with health professionals and people living with mental illness, who act as trainers as well as learners. This framework transforms traditional exchanges into mutual and collaborative learning experiences, strengthening the support and commitment of all learners.
	Process for establishing an innovative environment	Toney et al. (25) Crowther et al. (22) Doroud et al. (28)	Fosters a culture distinct from traditional health services, focused on education and coproduction. It encourages risk-taking and experimentation, and is fully integrated into the community.
	Balance of power process	Perkins et al. (1) McGregor, Repper & Brown (8) Shepherd et al. (24) Toney et al. (25) Crowther et al. (22) Toney et al. (5) Reid et al. (21) Doroud et al. (28)	Fosters reciprocal relationships between all learners by reducing power barriers and stigmatization.
	Self-determination development process	Perkins et al. (1) Sommer, Gill & Stein-Parbury (19) Toney et al. (25) Crowther et al. (22) Toney et al. (5) Reid et al. (21) Doroud et al. (28)	Fosters hope and self-determination. Learners are free to pursue the learning they want and develop the skills they consider important.
Principles	Formulated as general concepts, principles influence how we think about and develop training courses within RCs.		
	Personalised educational approach	Perkins et al. (1) McGregor, Repper & Brown (8) Perkins, Repper (23) Toney et al. (25) Crowther et al. (22) Toney et al. (5)	An educational approach to recovery that moves away from the therapeutic approach by enabling people to equip themselves and develop new skills. This approach focuses on the achievement of personalized learning objectives and the development of a learning plan in which learners make their own training choices. RC staff can support learners in their choice of training courses and in the development of their learning plan.
	Integrated community approach	Perkins et al. (1) McGregor, Repper & Brown (8) Perkins, Repper (23) Shepherd et al. (24) Toney et al. (5) Reid et al. (21) Doroud et al. (28)	An approach that relies on partnerships between RCs, health services and community services. This aims to adapt training proposals and modalities to community needs, thereby supporting the transformative potential of the RC learning model within services.
	Collaborative coconstruction approach	Perkins et al. (1) McGregor, Repper & Brown (8) Perkins, Repper (23) Toney et al. (5) Thompson et al. (27)	A collaborative approach where health professionals and people living with mental illness work together to design and deliver training courses. This coconstruction also takes place between learners, reinforcing mutual learning and innovation in educational programs, adapting training to the specific needs of learners. This involves the coproduction of knowledge, the cofacilitation of training courses and colearning.
	Approach embodying the recovery paradigm	Perkins et al. (1) McGregor, Repper & Brown (8) Sommer, Gill & Stein-Parbury (19) Toney et al. (5) Thompson et al. (27)	An approach embodying the recovery paradigm, where people, in recovery or not, are seen as individual citizens first and foremost. This approach refers to learner empowerment, hope, optimism, interconnection, and identity transformation.
	Inclusive approach prioritizing non-judgemental spaces	Muir-Cochrane et al. (20) Reid et al. (21) Thompson et al. (27)	An approach that provides a non-judgmental space where learners feel accepted, listened to and encouraged to participate actively.
	Diversity approach	Perkins et al. (1) McGregor, Repper & Brown (8) Perkins & Repper (23)	An approach where learners’ groups are composed of people in recovery, their relatives, professionals from health and community sectors, and other citizens.
	Approach recognising all types of knowledge	Perkins et al. (1) Perkins & Repper (23) Sommer, Gill & Stein-Parbury (19)	An approach that recognizes and values all types of knowledge equally, whether clinical, experiential or theoretical.
	Strength-based approach	McGregor, Repper & Brown (8) Perkins & Repper (23) Toney et al. (5)	An educational approach focused on the individual strengths. It encourages personal development by building on the strengths, abilities, and potential of each individual, focusing more the search for solutions than on problems.
	Ecological approach	McGregor, Repper & Brown (8)	An approach that considers the interaction of environmental, physical, social and cultural factors with the individual.
Operations	Presented in the form of infinitive action verbs, operations facilitate the concrete adoption of the principles.		
	Coproducing	McGregor, Repper & Brown (8) Perkins & Repper (23) Shepherd et al. (24) Sommer, Gill & Stein-Parbury (19) Crowther et al. (22) Toney et al. (5) Thompson et al. (27) Doroud et al. (28)	By sharing their clinical, experiential and theoretical knowledge, trainers and learners contribute to the construction of integrated knowledge.
	Creating a positive, interactive space	Perkins et al. (1) McGregor, Repper & Brown (8) Perkins & Repper (23) Crowther et al. (22) Toney et al. (5) Thompson et al. (27)	Trainers and learners contribute to creating a safe, positive and interactive space that offers a variety of learning resources.
	Colearning	McGregor, Repper & Brown (8) Sommer, Gill & Stein-Parbury (19) Thompson et al. (27)	Trainers and learners work together during sessions and learn from each other. They explore topics collectively to enrich each other’s knowledge.
	Cofacilitating	Crowther et al. (22) Perkins & Repper (23)	Trainers share the organization and running of sessions and workshops. They share roles and set up activities to optimize learner engagement and learning.

^1^“Transformative” here refers to the dynamic process through which RC principles are operationalized into meaningful outcomes for learners. This transformation involves changes in understanding, relationships, and self-perception, which may vary depending on individual and contextual factors. It is related to, but distinct from, transformative learning theory.

### 3.2 Identification of the key learning theories related to the RC learning model

**Figure 2:**
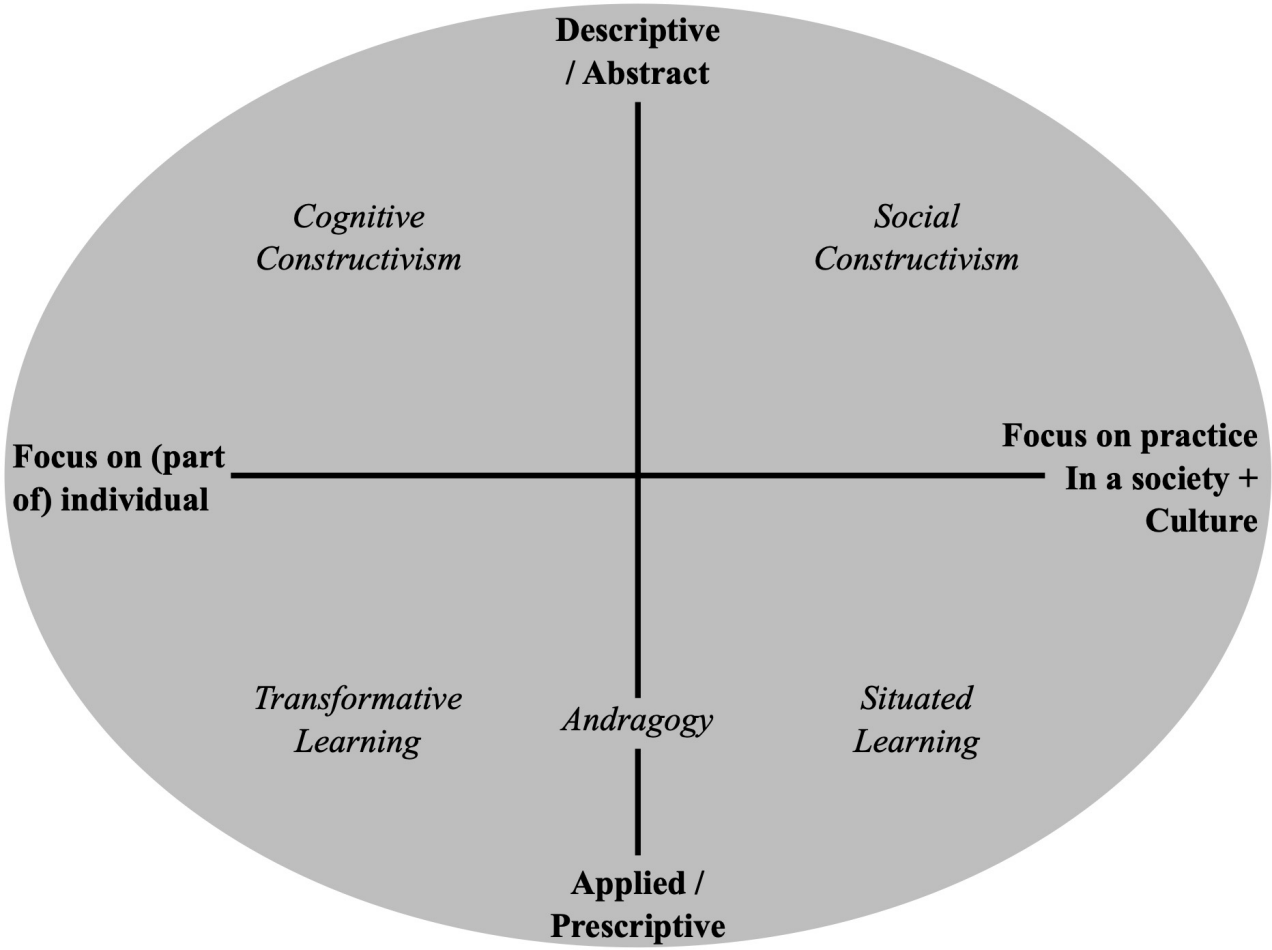
Positioning of learning theories retained according to the framework of Mann et al. (30).

The second corpus proposes complementary key learning theories relevant to the analysis of the RC learning model. According to Mann et al. (30) model, the key theories are divided into two axes (**Figure 2**): the individual/collective axis and the conceptual/practical axis. The individual/collective axis provides a frame of reference for positioning theories based on whether they emphasize learning from an individual perspective (psychological, cognitive factors, etc.) or a collective/social perspective (cultural, social, inspirational factors, etc.). Meanwhile, the conceptual/practical axis situates approaches according to whether they address the development of theoretical concepts or their concrete application (in teaching or other real-life situations).

On both individual and conceptual levels, cognitive constructivism (31–33) highlights the progressive development of mental structures through the learner’s actions and reflections. Similarly, cognitive dissonance theory (34, 35) underscores the role of cognitive imbalance as a driving force in the reorganization of knowledge. On an individual and practical level, transformative learning theory (36–38) stresses the importance of questioning frames of reference among adults, while andragogy (39, 40) emphasizes learners’ autonomy and prior experiences.

On both a collective and conceptual level, socio-constructivism (41–43) emphasizes the importance of coproducing knowledge through social interactions and the surrounding culture. On a collective and practical level, situated learning (44–46) concentrates on active participation within communities of practice and the importance of context. Lastly, the work of Steffe and Gale (32) enhances these perspectives by exploring the foundations of constructivism in greater depth. Goodrich (43) illustrates the diversity of interpretations within the Vygotskian legacy, confirming the complementary nature of these approaches.

This theoretical diversity highlights the significance of an integrative vision that takes into account learning dynamics at both individual and collective levels, as well as in both conceptual and practical dimensions. Consequently, an integrative vision can guide the analysis of the RC learning model. **Table 5** provides greater detail about these theories and their key elements, facilitating connections with the RC learning model.

**Table 5:** Presentation of the learning theories and their characteristics.

Theory	Key Element	Definition
**Social constructivism** Vygotsky (42) Berger & Luckmann (41) Goodrich (43)	Socio-constructivism, developed by Lev Vygotsky, is an approach to learning that integrates the principles of constructivism by adding a relational dimension. It emphasizes the central role of social interaction in cognitive development. With this approach, learning is a process structured by the many social interactions individuals experience in their environment. Thus, the construction of knowledge, while rooted in personal experience, is fundamentally influenced by social frameworks.	
	Social interaction	An essential element of learning where cognitive development results from interactions with more experienced people. Through these interactions, individuals internalize and transform observed actions into cognitive tools, building their knowledge through a process of social coconstruction.
	Zone of Proximal Development	It consists of the gap between what a learner can achieve on their own (their current level of development) and what they can achieve with the help of a more experienced adult or peer^1^ (their development potential). The ZPD represents the space where learning is optimal. It is where the individual can develop new skills with guidance. In this zone, learning is an active process in which the learner participates in tasks slightly above their current level, leading to progress.
	Scaffolding	The assistance provided in the ZPD can take the form of advice, demonstration or collaboration, facilitating the development of skills that later become autonomously mastered abilities. Thanks to the mediation of others, what is initially a skill observed, imitated and guided by others in a social context gradually becomes internalized by the learner.
	Social Construction of reality	Reality is a social construct; it is not objective but subjective. Individuals actively participate in constructing their reality rather than being passive vessels. Language, social interactions, and symbols all play a role in shaping this reality.
	Habitualization	The process by which a frequently repeated action becomes fixed in a pattern of behavior, enabling it to be reproduced later in the same way and with minimal effort.
	Institutionalization	A phenomenon occuring when habitual actions become shared and recognized patterns within a social group. These patterns mutually define the type of actions expected and the type of actors expected to perform them, thus forming an institution.
	The social stock of knowledge	Encompasses all the knowledge shared within a society, structured according to its general relevance or specificity to particular roles. The social stock of knowledge includes general knowledge that “everyone” possesses and more complex and specialized knowledge that pertains to certain professions or groups. Due to the division of labour, specialized knowledge tends to grow faster than general knowledge, creating fields of expertise that are increasingly complex and difficult for the uninitiated to access. This unequal distribution of knowledge influences perceptions and interactions within society.
	Development of a common language	Language can be defined as an objective reservoir of accumulated meanings and experiences, which it preserves over time and passes on to subsequent generations. It characterizes experiences by classifying them under broad categories, giving them a meaning shared by the individual and the community. From the point of view of social constructivism, language is the fundamental starting point for understanding reality, transcending perspectives centred on the external world or the individual mind.
**Cognitive constructivism** Piaget (31) Steffe & Gale (32) von Glasersfeld (33)	Cognitive constructivism, developed by Jean Piaget, is a learning theory emphasizing how individuals actively construct their understanding of the world. According to this approach, learning is an individual process in which knowledge develops through experimentation, assimilation, and the accommodation of new information into existing cognitive structures. Cognitive development is understood as a progressive process, marked by distinct stages where the learner continually adjusts their mental schemas in response to their experiences.	
	Assimilation	Cognitive process by which an individual integrates new experiences or information by associating them with existing behavioral or cognitive schemas. This mechanism enables the child to understand and react to new situations by applying pre-existing knowledge. Assimilation thus helps maintain continuity in understanding the world, while enriching the individual’s cognitive schemas.
	Accomodation	Cognitive process by which an individual modifies or reorganizes their existing behavioral or cognitive schemas to adapt to new experiences or situations that cannot be understood through current schemas. This mechanism is observed when a child realizes that his usual actions are not adapted to a new situation, and that they must adjust them to achieve their goal. This adaptation enables the individual to develop new cognitive schemas that are better adapted to their reality.
	Equilibration	Process by which an individual regulates their cognitive structures to maintain a balance between assimilation (integration of new information into existing schemas) and accommodation (modification of schemas in response to new information). This balancing process allows for knowledge adjustment and promotes harmonious cognitive development.
	Cognitive dissonance Festinger (34) Aronson (35)	Cognitive dissonance is not treated here as a full learning theory, but as a complementary concept from cognitive constructivism that sheds light on the role of cognitive conflict in learning. This concept describes the psychological tension experienced by an individual faced with conflicting beliefs, attitudes, or behaviors. This tension arises from the inconsistency between these elements. It can be exacerbated by factors such as the importance of decisions, the appeal of unchosen alternatives, and the degree of difference between options. Although cognitive dissonance does not directly stem from cognitive constructivism, it is related to it by potentially triggering a reorganization of cognitive schemas, a central tenet of constructivism. Both concepts highlight the role of cognitive conflicts in driving intellectual development and adjustment.
	Reduction	Cognitive dissonance reduction is a process by which an individual seeks to alleviate psychological discomfort resulting from contradictions between their beliefs, attitudes, or behaviors. According to Festinger, this can be achieved by modifying behavior, reinterpreting space to justify actions, or adding new cognitions that mitigate inconsistency. Individuals also use specific strategies to reduce dissonance after a decision, such as reinforcing the attractiveness of the chosen alternative, diminishing that of the rejected alternative, or identifying similarities between options to minimize discomfort. The overall goal is to restore internal coherence.
	Social Support	A crucial element in reducing cognitive dissonance by reinforcing the validity of an individual’s beliefs and behaviors. Support from others helps minimize dissonance, reducing feelings of isolation and providing social reinforcement that facilitates the adjustment of beliefs or behaviors.
	Logical inconsistency	Refers to internal contradictions that arise between different beliefs or between beliefs and experiences. It manifests in various forms: direct contradiction, conflict with cultural norms, opposition between specific and general beliefs, or contradiction with experience. Underlying cognitions, or deep beliefs, play a central role in influencing how individuals perceive and rationalize these inconsistencies, which can either aggravate or mitigate cognitive dissonance.
	Resistance to change	A phenomenon in which individuals refuse to adapt their behaviors or perceptions, despite cognitive dissonance. This resistance is reinforced by pre-existing commitment, perceived benefits, fear of the unknown, social pressures, and attachment to established beliefs, which provide cognitive stability and reduce motivation to change.
**Transformative learning** Mezirow (36) Mezirow (37) Hoggan (38)	Transformative learning is a holistic and dynamic process in which individuals revise and modify their frames of reference, including beliefs, habits of thought, and perspectives. Catalyzed by experiences such as disorienting dilemmas, this process involves critical reflection, dialogue, and affective engagement, enabling learners to question and reconstruct their prior meaning-making structures. Transformative learning fosters greater openness, inclusivity, reflexivity, and capacity for perspective-taking. It ultimately supports deeper personal growth, greater autonomy, and more adaptive and socially responsible action in complex contexts. As a metatheoretical construct, it integrates cognitive, emotional, and relational dimensions of learning and transformation.	
	Learning	The process of using a previous interpretation to develop a new or revised one to make sense of an experience and guide future actions. Learning occurs in four ways: through the elaboration of existing frames of reference, the development of new frames of reference, the transformation of perspectives, and the modification of habits of thought. These changes allow for a more profound adaptation and understanding of the world.
	Reflexive learning	Involves confirming, expanding or transforming perspectives of meaning through the evaluation and re-evaluation of underlying assumptions. This process becomes transformative when assumptions are identified as distorted, inauthentic or invalid. Reflexive learning involves open, rational dialogue that is empathetic and receptive to different points of view, enabling the testing of one’s beliefs and the exploration of alternative perspectives to foster profound, critical learning.
	Transformation	A process of revising fixed structures of meaning through the reconstruction of dominant narratives. This process involves a profound change in how individuals interpret and understand their reality. Over time, this transformation can become a lasting frame of reference, influencing how a person approaches new situations and interprets the world, thus adopting a new dispositional orientation.
	Personal experience	Refers to life experiences that provide a foundation for critical reflection and exchange. These experiences encourage individuals to question their beliefs and ground their learning in personal context, fostering a transformation of perspectives.
	Empowerment and personal growth	A process through which individuals develop a deeper understanding of themselves and their place in the world, leading to more autonomous and informed decision-making while promoting increased self-awareness and personal growth.
	Disorienting dilemma	An experience or event that challenges a person’s existing beliefs, assumptions, or worldview, creating cognitive and emotional discomfort that prompts reconsideration and learning.
	Critical reflection	The process of critically examining one’s assumptions, beliefs, and values, often triggered by a disorienting dilemma, to foster deeper understanding and personal transformation.
**Andragogy** Knowles (39) Merriam & Bierema (40)	Andragogy is a discipline that develops teaching methods adapted to adults, taking into account their experience, needs and autonomy. It values their ability to direct their own learning, and uses their experiences as a basis for acquiring new knowledge. Andragogy aims to create a learning space that respects and encourages the maturity and independence of adult learners.	
	Self-concept	Refers to adults’ perception of their ability to direct their own learning. They prefer to take an active and influential role in the process, choosing not only the content to be learned, but also the learning methods. This concept highlights the importance of creating spaces that value learners’ autonomy and independence, allowing them to control their own development and fully engage in their progress.
	Personal experience	The set of personal and professional life experiences of adults, which constitutes a key resource in their learning. These experiences serve as a foundation for the development of new concepts, linking learning to real and meaningful contexts. By integrating these experiences into the educational process, learning becomes more relevant and concrete, facilitating critical thinking and the practical application of new knowledge in everyday or professional situations.
	Readiness to Learn	Refers to the readiness of adults to engage in a learning process when it meets immediate or relevant needs in their social, professional, or personal roles. Adults are particularly receptive when the content is directly applicable to solving concrete problems or improving their effectiveness in these roles. This readiness to learn is therefore reinforced by the perceived relevance of the learning and its immediate usefulness in their daily lives.
	Motivation	A set of forces that inspire engagement in the learning process. Motivation is often intrinsic, rooted in personal or professional goals such as the pursuit of self-development or skill improvement. However, external factors, such as recognition or tangible rewards, can also play a role. To sustain adult learners’ motivation, it is essential to create an environment that enhance their sense of self-efficacy and emphasize the relevance of the skills being developed.
	Active space of learning	A space of learning that is dynamic, stimulating, and inclusive, designed to address the needs of learners, particularly adults. It fosters a sense of security and trust while encouraging active participation. In this space, learners are autonomous, can exercise control over their learning process, and are fully involved, fostering deeper engagement and collaborative knowledge exploration. This type of space values interaction and critical thinking while supporting both personal growth and skill development.
**Situated Learning** Lave & Wenger (44) Brown et al. (45) Greeno (46)	Situated learning occurs within authentic social and practical contexts, in a community of practice. Individuals evolve from novices to experts by gradually participating in collective activities, developing tacit knowledge and a new identity within the group. Anchored in real-life situations, this learning is inseparable from social interaction and context, facilitating the transfer of skills into practice. The reification of knowledge in the form of artifacts contributes to its dissemination within the community.	
	Identity transformation through peripheric Learning	A process of identity transformation through which an individual evolves from novice to expert within a community of practice. The process occurs through individuals’ interaction with peers at varying levels of expertise. Individuals discuss, listen to, and observe more experienced community members before taking action. This process involves gradual immersion, where the individual begins with simple peripheral tasks under the guidance of experienced members. Over time, this fosters a sense of belonging to the group, enabling novices to adopt cultural codes, share values, and shape their identity. By progressively participating in collective practices, the individual develops their identity, role, and belonging to the group, ultimately transforming their perception of themselves and becoming fully recognized members.
	Community of practice	A group of people sharing common interests who interact regularly to deepen their expertise and develop skills. It is based on three main elements: a shared area of interest, social relationships between members, and common practices. Learning occurs through active participation in group activities, where everyone contributes through their individual experience. This concept, developed by Wenger, emphasizes collaborative learning and ongoing engagement in collective practices.
	Learning in authentic context	A learning process that takes place in real or simulated spaces, reflecting the conditions in which knowledge and skills will be used. By anchoring learning in concrete situations, this approach facilitates the transfer of skills to practical contexts. Unlike decontextualized teaching methods, it encourages active participation in authentic social practices, allowing learners to mobilize their knowledge in a relevant way and acquire a deeper understanding of the disciplines studied.
	Tacit knowledge	Unformalized knowledge developed through experience and immersion in the practices of a community. It encompasses skills, behaviors, and norms that are not explicitly taught but are transmitted through daily actions and interactions. This type of knowledge is essential for becoming a fully integrated member of a community of practice and is often challenging to articulate or formalize, eluding traditional teaching methods. It primarily develops through observation and active participation.
	Réification	Process through which experiences and practices are crystallized into tools, documents, concepts, or symbols. These artifacts enable members to share complex practices in a more tangible way and to transmit knowledge to others, including novices.

^1^Social and cognitive constructivism stem from Vygotsky’s and Piaget’s seminal work on child development. In the context of this study, we can draw parallels between children and learners, as children have often been studied from the perspective of their learning processes. In this view, adults are seen as experienced transmitters of knowledge or trainers. To preserve the authors’ original terminology, the words “children” and “adults” are used in this table, but these terms will not be repeated throughout the rest of the text.The light gray shade intend to visually distinguish each theory with its corresponding definition.

### 3.3 Analysis of the connection between the RC learning model and key learning theories from three philosophical perspectives

This section provides a philosophical analysis of the RC learning model from three perspectives: epistemological, ethical, and political. For each, three questions were formulated, enabling connections between the RC learning model and key learning theories.

#### 3.3.1 Epistemological analysis

Epistemology, which relates to how knowledge is produced and learned, guides the analysis through the prism of three questions: 1. What is considered knowledge? 2. How is new knowledge developed and transmitted? 3. What are the knowledge development objectives in the RC learning model?

##### 3.3.1.1 Epistemological posture of the RC learning model

*What is considered knowledge?*

In the RC learning model, knowledge is viewed as pluralistic and inclusive, adopting an approach that integrates and values three main types of knowledge: clinical, experiential, and theoretical. The fundamental principle here is the “approach recognizing all types of knowledge,” as it places the different types of knowledge on equal footing. Theoretical knowledge, being abstract and formal, is derived from academic disciplines and provides a general framework for understanding concepts, models, and approaches in mental health. Clinical knowledge, being more pragmatic and contextual, relies on the experiences of health professionals and is developed through direct interactions with patients in clinical settings. Finally, experiential knowledge, centred on the lived experiences of individuals, is particularly valuable for providing unique and subjective perspectives on the human condition, which are often overlooked in more traditional settings.

The mechanism of action, described as the “process of emergence of different relationships,” actively blurs the distinctions between various types of knowledge. It fosters the establishment of spaces where trainers and learners coproduce integrated knowledge, merging different types of knowledge and facilitating mutual learning. In this sense, the RC learning model is grounded in a socioconstructivist vision, where knowledge is coproduced based on the context, acknowledging the value of diverse knowledge. This mechanism of action also challenges traditional hierarchies of knowledge by promoting collaborative exchange. The operations of “coproducing” and “colearning” illustrate this dynamic, as learners share their insights from lived experiences, theory, and clinical practice to forge a more holistic understanding of various knowledge domains. The collaborative framework established in RC learning spaces is further reinforced by the “collaborative approach,” which encourages a collective production of knowledge in which everyone plays the dual role of both trainer and learner. This active coproduction supports the recognition of the intrinsic value of each type of knowledge and its complementarity, ultimately fostering a more nuanced and comprehensive understanding of mental health issues.

*How is new knowledge developed and transmitted?*

The RC learning model aims for a learning process that is both autonomous and collective, supported by various mechanisms of action, approaches, and operations. The mechanism of action known as the “self-determination development process” plays a central role in emphasizing learners’ freedom to choose the learning objectives they wish to pursue. This is connected to the “personalized educational approach,” which positions the learner actively to identify their own learning objectives based on their needs and interests. Personalized support also aids learners in constructing their learning paths. In this context, knowledge development is considered a coproduction process, as illustrated by the “collaborative coconstruction approach.” This approach fosters the active engagement of learners and trainers in a dynamic where they learn from one another. Thus, knowledge development is not restricted to unidirectional transmission but instead involves a dynamic and continuous exchange (“colearning” operations).

Furthermore, the learning environment plays a crucial role in this process. The mechanism of action, referred to as the “process for establishing an innovative environment,” creates a framework that fosters experimentation and risk-taking, promoting the exploration of new knowledge forms. This interactive space, enabled by the “creating a positive, interactive space” operation, supports knowledge development that centres on the engagement of active learners and a non-hierarchical approach to learning.

*What are the knowledge development objectives in the RC learning model?*

The development of new knowledge within the RC learning model pursues several key objectives. Firstly, it seeks to promote the personal transformation of learners. The mechanism of action, “process of personal development and transformation,” illustrates how knowledge development enables individuals to reshape their self-perception and abilities, facilitating a shift towards a more positive, normalized self-image. This process correlates with the “approach embodying the recovery paradigm,” where empowerment, optimism, and identity transformation are essential.

This transformation is not limited to individuals in recovery but extends to all learners, including health professionals. By integrating learners’ experiential knowledge, professionals are encouraged to reconsider their perspectives and practices, thereby enriching their professional approach. Through the principle of an “inclusive approach prioritizing non-judgmental spaces” and the “coproduction” operation, the RC learning model fosters egalitarian relationships where each individual, regardless of their background, can participate in the co-production of knowledge. Consequently, RC learning spaces are grounded in the value of epistemic justice, advocating that all discourses and experiences can be legitimate sources of knowledge. Egalitarian epistemic spaces can help deconstruct traditional power dynamics, thus contributing to the reduction of implicit prejudice and the fight against stigmatization in the therapeutic relationship.

Ultimately, the development of knowledge aims to drive systemic transformation. By revising individuals’ perceptions and beliefs, the RC learning model sensitizes its learners to the social and institutional issues surrounding mental health. In this manner, learning modifies individual trajectories and the structures and systems in which they develop. This aligns with the “community-integrated approach,” which seeks to adapt training courses to the needs of community services and influence the culture of mental health services. These spaces also rely on the mechanism of action, referred to as the “process for establishing an innovative environment,” which creates a framework that encourages experimentation and coproduction, enabling changes to be incorporated into institutional practices. In the RC context, learning is not confined to personal or individual effects but aspires to influence power structures and systems by promoting inclusion, innovation, and coproduction in the mental health field.

##### 3.3.1.2 Connections with key learning theories

The RC learning model exists at the intersection of several key learning theories, such as socio-constructivism, cognitive constructivism, situated learning, andragogy, and transformative learning. What constitutes knowledge—be it theoretical, clinical, or experiential—aligns with Berger and Luckmann’s (41) concept of the ‘social stock of knowledge’ and Vygotsky’s (42) idea of collective knowledge construction. These authors argue that the “coproducing” mechanism and the “collaborative coconstruction approach” reflect learning through social interaction, encouraging a continual reassessment of traditional hierarchical structures. Mechanisms of action such as the “emergence of different relationships,” which blur hierarchical distinctions in knowledge, can also be likened to Vygotsky’s Zone of Proximal Development (ZPD), where social interactions play a crucial role in facilitating mutual learning and exceeding individual capabilities. However, it is important to note that, within the ZPD framework, hierarchical structures between novice and expert persist, even as these interactions foster shared learning.

The process of developing new knowledge in the RC learning model illustrates the autonomy valued in andragogy (39) and reflects the dynamics of cognitive constructivism (31), where assimilation and accommodation evolve through active interaction. Furthermore, cognitive constructivism (31) emphasizes the progressive elaboration of mental structures based on experience. This process is evident in the dynamics of RC, where knowledge is cultivated through the active, reflexive participation of learners, as shown by the operations “colearning” and “cofacilitating.” The RC learning model also incorporates the principles of cognitive dissonance (34, 35), which are observable when learners from diverse backgrounds—such as individuals in recovery, health professionals, family members, students, among others—confront divergent perspectives and experiences, creating discomfort that encourages them to revise their beliefs and reorganize their cognitive schemas. The contextual and social dimension of situated learning (44) is highlighted in the “process for establishing an innovative environment” and “creating a positive, interactive space.”

Finally, the model aims to prompt both personal and systemic transformation, aligning with transformative learning theory (36–38). The “process of personal development and transformation” and the “balance of power process” not only redefine the individual framework but also challenge and recast institutional structures, thereby establishing a direct link with the critical dialogue approach and the principle of “reflexive learning”.

#### 3.3.2 Ethical analysis

Ethics is a philosophical discipline whose object encompasses the values and principles that should govern living together. This philosophical perspective is approached from two questions: 1. What values underlie the RC learning model? 2. How are these values embodied and transmitted?

##### 3.3.2.1 Ethical posture of the RC learning model

*What values underlie the RC learning model?*

The RC learning model is founded on a set of fundamental values guiding pedagogical and relational practices, including strict equality of knowledge and people, coproduction, benevolence, authenticity, empowerment, inclusion and respect for people and learner autonomy. The RC learning model endorses an ethical posture that shifts from a prescriptive model to one that favours collaborative learning centred on each individual’s experience. Another key aspect representing a core value of the RC learning model is the “balance of power process”. This mechanism of action aims to deconstruct the hierarchies between trainers and learners by promoting an egalitarian relationship. In this sense, the RC learning model adopts a relational ethic, where every actor in the educational process is essential. This approach is reinforced by the “coproducing” operation, which encourages the collaborative creation of educational content between professionals and people in recovery. Unlike traditional learning models, which can convey a hierarchical, top-down logic, the RC learning model is based on mutual engagement and horizontal knowledge sharing.

Rooted in the principle of non-judgment, the values of benevolence and respect lie at the core of the RC learning model experience. These values are reflected in the “inclusive approach prioritizing non-judgmental spaces,” which ensures an environment where everyone feels valued in their learning journey, irrespective of their background, status, or life experiences. This ethical commitment also manifests in the “creating a positive, interactive space” operation, which helps fostering an atmosphere conducive to trust and genuine dialogue.

*How are these values embodied and transmitted?*

The embodiment of the values of the RC learning model is rooted in an experiential and collaborative approach, where ethical principles are not merely stated but integrated into daily practice. The mechanism of action, or the “process for establishing an innovative environment,” plays a crucial role in creating a learning space that distinguishes itself from traditional healthcare institutions, where learners are not simply regarded as “patients” or “professionals” but are viewed as active learners. This process promotes the active appropriation of the RC learning model’s values by learners, who emerge as agents of transmission. The ethics of the RC learning model are also reflected in participatory pedagogy, which encourages interactions through the “colearning” operation. By highlighting mutual learning, this approach deconstructs traditional power dynamics and fosters an ethic of dialogue, where everyone plays an active role in generating collective knowledge.

The cofacilitation of RC courses reflects a commitment to role equality and shared contributions. The « cofacilitating » operation aims to balance the perspectives of individuals from different backgrounds and experiences. Trainers embody the principles of the RC learning model through their posture and interactions, acting as key agents in transmitting its values. Their open, non-hierarchical stance fosters cooperation and mutual respect among learners. Finally, the transmission of values involves concretely experiencing the principles of the RC learning model in a safe and inclusive space. Ultimately, value transmission occurs through the lived experience of these principles in a safe and inclusive environment. The ‘creating a positive, interactive space’ operation is central to supporting meaningful learning and personal transformation.

##### 3.3.2.2 Connections with key learning theories

Following this ethical analysis, the RC learning model is grounded in various theoretical approaches including andragogy, social constructivism, transformative learning, and situated learning. The “personalized educational approach” is rooted in andragogy (39), which values autonomy and the active construction of an individualized educational journey. Regarding the “balance of power process” mechanism, it aligns with social constructivism (41, 42), which emphasizes interactions and the coproduction of knowledge to challenge traditional hierarchies and foster collaborative knowledge creation. Moreover, the transformative goal of the RC learning model, demonstrated by the mechanism of action “process of personal development and transformation,” aligns with the principles of transformative learning (36) by underscoring the significance of critical experiences for re-evaluating beliefs and biases, as well as redefining identity.

In addition, the “co-learning” operation integrates principles of social constructivism and situated learning (44) by fostering a proximal zone of development (PZD) in which peer exchange supports the concrete appropriation of the mental health recovery paradigm. Colearning is reinforced by the operation “creating a positive, interactive space,” which establishes a safe environment conducive to the emergence of new learners’ identities, and by “cofacilitating,” which promotes horizontal, participatory knowledge exchange through empowerment and critical awareness. By valuing learners’ lived experiences as legitimate knowledge, the RC learning model challenges epistemic inequalities and affirms the credibility of marginalized knowledge. Its inclusive approach and commitment to egalitarian recognition support the intrinsic worth of each individual, fostering both moral and social recognition. In this context, learning becomes not only a cognitive process but also an act of social affirmation that promotes dignity and personal growth.

#### 3.3.3 Political analysis

Politics in philosophy examines the concepts of governance, social justice, rights, freedom, and authority, studying how individuals and organizations interact, influence society, and exercise or challenge power based on ethical values. The following questions were raised as part of this study: 1. How is power distributed among people in learning spaces? 2. What strategies are employed within the RC learning model to promote equality among individuals, including learners?

##### 3.3.3.1 Political posture of the RC learning model

*How is power distributed between people in learning* sp*aces?*

In the learning spaces of the RC learning model, efforts are made to reduce power relations through various mechanisms, approaches, and operations. For example, the mechanism known as the “balance of power process” aims to minimize hierarchies by promoting reciprocal relationships among learners, including individuals in recovery, healthcare professionals, and other learners. The “collaborative coconstruction approach” seeks to ensure equal participation for those with lived experience and healthcare professionals in the design, facilitation, and delivery of RC courses. This approach recognizes and values diverse clinical, experiential, and theoretical knowledge, thereby strengthening mutual learning. More specifically, the “cofacilitating” operation empowers trainers to jointly determine learning content and activities and share roles during sessions. This distribution of responsibilities among trainers helps to dismantle power barriers and serves as a model for learners, who, in turn, influence the learning process as coproducers. *What strategies are used within the RC learning model to promote equality among individuals, including learners?*

To promote equality, the RC learning model relies on strategies designed to transform learning dynamics and foster equal participation among all learners. Coproduction is central to the RC learning model, enabling all learners to contribute equally to knowledge creation. By valuing diverse forms of knowing within trainer dyads and learner groups, the model aligns with the recognizing all types of knowledge approach and challenges traditional hierarchies of legitimacy and credibility.

The “inclusive approach prioritizing non-judgmental spaces” aims to create a welcoming environment where learners and trainers feel heard and accepted, thus enhancing equality in participation during learning activities. The mixed composition of learner groups enriches perspectives and encourages open dialogue, contributing to a more inclusive learning experience. The “strength-based approach” is integral to this framework, as it values learners’ skills and personal resources, promoting empowerment and a more equitable distribution of learning opportunities. These strategies, which emphasize collaboration, inclusion, and empowerment, strive to cultivate a learning environment where equality is central.

##### 3.3.3.2 Connections with key learning theories

Following this political analysis, the RC learning model proposes creating learning spaces designed to mitigate power relations through mechanisms such as the “balance of power process” and the operation of “cofacilitating”. By promoting reciprocal relationships and power-sharing between learners and trainers, these mechanisms illustrate a conscious reduction of hierarchies in accordance with the principles of social constructivism and the element of scaffolding (42, 43), where temporary help is offered to allow the gradual development of new skills.

The sharing of roles between trainers and learners reduces hierarchies and facilitates the creation of zones of proximal development (ZPD), where each can grow by receiving support tailored to their needs. Similarly, equality is promoted through strategies such as “coproduction,” which, by valuing diverse knowledge, aligns with the “approach recognizing all types of knowledge” and the “collaborative coconstruction approach,” ensuring an equal place for every individual. The application of the “inclusive approach prioritizing non-judgmental spaces” and the “Strength-based approach” contributes to creating a welcoming environment and enhancing individual empowerment (39, 40).

Moreover, the political dimension of transformative learning theory is particularly resonant with the RC learning model. Beyond fostering individual empowerment, transformative learning emphasizes the development of critical consciousness and the ability to question and act upon social, cultural, and institutional structures (38). The RC learning model’s aligns directly with these objectives. Thus, transformative learning theory offers a valuable lens for understanding how RC not only promotes personal growth but also supports broader social change and democratization of knowledge.

Finally, integrating these dynamics into a transformative and andragogical learning perspective reflects a desire to reshape trainers’ and learners’ identities within a community of practice. As defined by Lave and Wenger (44), such communities combine experience and active participation to deconstruct power relations and promote an authentic, horizontal transmission of knowledge.

## 4 Discussion

This article aimed to characterize the Recovery College learning model and identify its connections to key learning theories through theoretical and philosophical analysis. The study highlighted the approaches, operations, and mechanisms of the RC learning model, engaging them in dialogue with established learning theories. The discussion section synthesized the main contributions of the study, emphasizing the innovative nature of the RC learning model and its epistemological, ethical, and political implications. Finally, the strengths and limitations of the study are addressed.

### 4.1 A model based on principles, operations and action mechanisms

The corpus of texts on the RC learning model has revealed some terminological variability concerning its definition and characterization. This study proposes a definition and characterization based on the existing literature on RC and builds upon the work of Perkins & Reppers (1, 23) and Toney et al. (25), who explored the mechanisms of action behind the RC learning model’s effectiveness. This study adopts a multilevel approach, in line with the rationale presented by Astbury and Leeuw (13). These authors argue that the mechanisms of action function at different levels of transformation, offering insight into how an educational program produces its effects. In the case of RC, this approach facilitates the connection between the fundamental principles of the learning model, the operations carried out in the courses, and the outcomes for learners.

The analysis thus reveals three main levels in structuring the learning model. On the first level, the principles influence the way in which courses and learning spaces are conceived and develop within each RC. A second level, operations, operationalizes the principles into concrete actions and offers more precise keys on how to build and conduct RC courses. The third level is transversal and comprises the mechanisms of action. They play a fundamental role in linking the principles to the outcomes through operations. They function as transformative processes that enable learners to develop and transform knowledge, turning it into tools for recovery. These mechanisms of action include the process of personal and transformational development, the emergence of new relationships, the establishment of an innovative educational space, the rebalancing of power relations and the development of self-determination. They show how implemented strategies shape learning trajectories and foster lasting change. By integrating these three levels of analysis, the study provides a clearer understanding of the RC learning model and the processes within collective mental health learning spaces.

### 4.2 A model rooted in educational theories

This study underscores the alignment of the RC learning model with foundational learning theories across various educational traditions, detailing the operational mechanisms that substantiate its effectiveness and theoretical grounding.

Socio-constructivism represents a major influence. Inspired by the work of Vygotsky (42), it highlights the importance of social interactions and the coproduction of knowledge. Knowledge is constructed through contextualized interactions, where each learning experience is unique and leads to a personal construction of knowledge. The RC learning model, with its emphasis on combining diverse forms of knowledge and fostering collaboration among individuals in recovery, health professionals, and other citizens, aligns with this perspective. Cognitive constructivism, as defined by Piaget (31), helps explain how RC learners adapt and adjust their cognitive schemas in response to new knowledge. Through assimilation and accommodation, they integrate clinical, experiential, and theoretical knowledge, thus promoting a progressive transformation of their understanding and perceptions. While theoretical knowledge is often readily assimilated, practical and experiential knowledge, when considered collectively, challenges our existing understandings, prompting the necessary accommodation and thus engaging learners in a more complex learning process that involves the deconstruction of prior assumptions.

Mezirow’s (36, 37) transformative learning aligns well with the RC learning model, which aims to challenge learners’ frames of reference and empower them to redefine their life trajectories through critical reflection on past experiences and prior knowledge. The RC model is also aligned with principles of andragogy (39). This theory emphasizes learner autonomy, self-directed learning, and the consideration of prior experiences as essential resources for developing new knowledge. By providing a flexible learning framework where learners can select their educational paths based on their needs and interests, RC effectively illustrates the principles of adult education.

Finally, situated learning, as theorized by Lave and Wenger (44), is reflected in the way the RC learning model creates communities of practice where learning takes place within a social framework anchored in the learners’ reality. Recognizing the value of lived experiences, involving people in recovery in the cofacilitation of courses, and supporting co-learning between learners all contribute to building knowledge directly applicable to their daily lives. Additionally, the possibility of long-term engagement with RC members fosters ongoing mutual influence through multiple learning experiences and interactions, thus enriching and continuously reshaping individual and collective knowledge.

As Pawson and Tilley (14) argue, social interventions such as RC necessitate theoretical pluralism, since no single explanatory framework can capture their full complexity and context-dependence. Embracing multiple theoretical perspectives allows us to better understand the diverse mechanisms and outcomes of the RC model, while also highlighting areas where further theoretical integration and critical dialogue may be beneficial. It is equally important to acknowledge that not all dimensions of these learning theories fully align with or capture the specificities of the RC learning model, which operates at the intersection of individual, relational, and systemic change processes that often exceed the explanatory focus of any single theory.

In short, the RC learning model embodies several complementary learning approaches and theories. This study provides a better understanding of the model’s deep educational roots, which explain its effectiveness. It reinforces the conceptual validity of the RC learning model and its relevance to the field of health education.

### 4.3 A model with epistemological, ethical and political implications

#### 4.3.1 An inclusive and transformational pedagogy

The RC learning model differs from traditional educational models by its inclusive, collaborative approach centred on the colearning of learners from diverse backgrounds. Unlike traditional educational models that rely on top-down knowledge transmission, RC offers a horizontal learning model where each learner is both a learner and a coproducer. This model blurs boundaries between teachers and students, professionals and beneficiaries, by fostering a learning dynamic grounded in recognition of lived experience.

One of the fundamental elements of the RC learning model is the recognition of epistemic plurality, where knowledge is not exclusively derived from academic or professional expertise but also from lived experience. This perspective counteracts the dominant epistemology in educational and medical institutions, which tends to privilege formal knowledge and marginalize other forms of understanding (47). By placing clinical, experiential, and theoretical knowledge on an equal footing, the RC learning model challenges the traditional hierarchy that grants greater legitimacy to academic knowledge and is part of a movement in health education (30, 48).

In the context of RC, epistemic pluralism is recognized through the coproduction of learning, an approach inspired by social constructivism (42). For instance, a person with experience of mental illness can teach alongside a health professional, thereby promoting a sharing of perspectives and questioning of established norms. This dynamic facilitates a transformation of representations and practices, not only for the learners but for the professionals involved.

Overall, the RC learning model is based on the concept of epistemic justice (49). According to this philosopher, epistemic justice consists of two dimensions: testimonial justice and hermeneutic justice. It is achieved when both dimensions are upheld consistently. Testimonial justice pertains to the credibility given to an individual. When someone is recognized as a valid epistemic agent and their discourse is considered credible, their voice is acknowledged. Testimonial injustice occurs when a person’s discourse is unfairly dismissed. The RC learning model, which values and recognizes everyone’s voice, promotes the advancement of testimonial justice. Hermeneutic justice involves a person’s ability to articulate their experiences through existing knowledge. Hermeneutic injustice occurs when an individual cannot do so. Since the RC learning model is tied to the coproduction of knowledge, it incorporates learners’ experiences, allowing them to express their own perspectives. Consequently, the RC learning model serves as a learning environment grounded in the principle of epistemic justice.

#### 4.3.2 Profound ethical implications: a space for social and epistemic justice

The RC learning model is based on an ethical vision of education that goes beyond the simple development of knowledge to include dimensions of social justice and individual empowerment. In addition to enabling the development of new knowledge, the model seeks to transform relationships between individuals and foster learners’ empowerment.

The RC approach aligns with the principles of transformative learning (37), which focuses on questioning frames of reference and critically re-evaluating beliefs. The transformative aspect is central to empowerment strategies, especially in mental health, where individuals in recovery are often marginalized and perceived as passive recipients rather than knowledgeable actors (50). The RC learning model is dedicated to promoting learner self-determination. Instead of imposing a rigid educational framework, this model enables learners to create their own learning paths based on their interests and needs. This autonomy in learning fosters a sense of control and self-efficacy, both of which are crucial for mental health recovery.

This approach aligns with the ethics of care (51), which emphasize relationships rooted in attentiveness, reciprocity, and recognizing vulnerability as a strength. Within this framework, learning courses become a space of mutual support and social rehabilitation, where each individual can contribute based on their experiences and skills while maintaining a stance of openness, kindness, and respect.

As discussed, a key innovation of the RC learning model is its ability to address epistemic injustices (49), the dismissal or devaluation of marginalized people’s knowledge. By creating space for individuals to share and legitimize their stories, the model fosters understanding of diverse realities and transforms professional practices. Because epistemic injustices often precede other forms of social injustice (52, 53), such as distributive or occupational, this approach is ethically vital. Recognizing individuals as credible enables public policies to distribute more equally the social goods that support dignity, decency, and freedom from oppression (54).

#### 4.3.3 Political issues: towards a democratization of knowledge

The RC learning model is not confined to pedagogical transformation; it is also part of a broader political dynamic aimed at redefining power relations and democratizing knowledge. By relying on a collaborative and inclusive approach, RC challenges traditional hierarchies between clinical, experiential, and theoretical knowledge. Thus, it aligns with Freire (55) work on critical pedagogy, which denounces the oppressive nature of teaching where the student is simply a passive receptacle. From this perspective, RC proposes a more horizontal model in which everyone can actively contribute to the production of knowledge.

Politically, the recognition of experiential knowledge within the RC learning model also helps combat systemic discrimination, particularly sanism (56). By fully integrating individuals in recovery into RC courses and educational processes, the RC learning model provides an inclusive framework that redefines the standards of knowledge legitimacy and paves the way for reform in educational and clinical practices. This framework aligns with the goals of more participatory citizenship, where access to knowledge becomes a tool for empowerment and social transformation. Moreover, it is founded on anti-oppressive practices towards individuals with various mental health conditions. In this sense, the RC learning model serves as a relevant and powerful tool for fighting sanism, a system of oppression against individuals deemed ‘not of sound mind’ or living with mental illness.

Finally, the RC learning model goes beyond offering an alternative theoretical framework; it also aims to transform institutional practices by promoting pedagogical innovation and shaping public policies towards equitable, inclusive, and anti-oppressive strategies for individuals facing mental health challenges. For example, in the UK, Recovery Colleges have been explicitly integrated into national mental health policy through NHS England’s implementation frameworks (2), and their development is recognized as one of the ten key components of recovery-oriented services (1), illustrating their practical influence on system-level priorities. Advocating for an integrated approach within health services and communities aids in reconfiguring learning environments into spaces for collective empowerment. In this regard, the model acts as a genuine laboratory for democratic experimentation, where knowledge becomes a tool for combating inequality and a catalyst for sustainable social change.

### 4.4 Strengths and limitations

This study presents several strengths and limitations. First, some limitations should be noted. Due to its exploratory approach, this study relies on the interpretation of texts and the conceptual connection of the RC learning model with various learning theories. While this analysis offers an original perspective, it involves a certain degree of subjectivity in identifying theoretical connections. Furthermore, the corpus regarding learning theories relies heavily on foundational texts, whose concepts have developed and been reformulated in more recent works that are not fully considered here. The study could have also explored the potential tensions between the various theories utilized. Also, the absence of an explicit distinction between general and adult-learning theories limits the precision of the theoretical analysis for adult education contexts. Although these learning theories enhance our understanding of the RC learning model, they do not converge seamlessly. A more critical analysis would have refined the reflection and provided a clearer understanding of the theoretical challenges in articulating the RC learning model.

The strength of this article lies in its scientific contribution to understanding the RC learning model. It is based on a rigorous hermeneutic philosophical approach, allowing for an in-depth analysis of the model’s epistemological, ethical, and political foundations. By mobilizing concepts from education, philosophy, and mental health, it adopts an interdisciplinary approach that enriches reflection and emphasizes the epistemic and social justice issues related to integrating experiential knowledge. By identifying precise mechanisms of action, approaches, and operations and their connection to various learning theories also strengthens the legitimacy of the RC learning model.

## 5 Conclusion

The theoretical and philosophical analysis of the RC learning model demonstrates its richness and originality within the educational and mental health landscape, as well as its grounding in several inspiring learning theories. Through its approaches of coproduction of knowledge, recognition of lived experiences, and inclusive learning, the RC learning model is genuinely transformative. Its foundation in various learning theories illustrates its ability to redefine traditional frameworks for knowledge development. Beyond its epistemological implications, the RC learning model raises profound ethical and political issues, promoting epistemic justice and social equity, reducing power imbalances, and democratizing knowledge, as well as overturning the system of oppression known as sanism.

While RC is emerging as an innovative learning model, its implications extend far beyond the educational framework, fundamentally questioning the organization of mental health care and, more broadly, societal perceptions of mental illness and individuals with diverse mental health experiences. With its inclusive and collaborative approach, the model invites a rethinking of professional practices, the relationships between users and health professionals and even institutional structures. Therefore, a broader reflection is required on how to integrate this model into health systems to maximize its impact and ensure its sustainability. Its institutional grounding poses organizational and cultural challenges, necessitating adaptations to local contexts, greater recognition of expertise derived from experience, and an evolution of normative frameworks. Far from merely complementing traditional approaches, the RC learning model offers pathways for transforming public policies and fostering a more equitable, inclusive, and anti-oppressive vision of mental health. By placing experiential knowledge and coproduction at the center, it becomes a driving force for innovation and both individual and collective empowerment.

## Data Availability

The raw data supporting the conclusions of this article will be made available by the authors, without undue reservation.
